# Supplementary material for: Mixed Infections Unravel Novel HCV Inter-Genotypic Recombinant Forms within the Conserved IRES Region
Source: Viruses. 2024 Apr 3;16(4):560. doi: 10.3390/v16040560 (PMC11053413; doi:10.3390/v16040560)
Supplement: Supplementary file 1 [file viruses-16-00560-s001.zip › viruses-2904069-supplementary.pdf]

Supplementary Materials

Supplementary Table S1. Demographic characteristics of patients enrolled in this study and virological parameters

Sample	Sex	Age (years)	Viral Load (log10) [a]	Antiviral therapy [b]	Response to treatment [c]
598	M	53	5.78	PR	NR
624	M	21	6.80	ND	ND
955	F	65	6.61	PR	ND
982	M	45	5.08	PR	NA [d]
990	M	40	6.71	PR	SVR
991	M	48	7.16	ND	ND
996	F	72	5.62	ND	ND
1011	M	31	6.48	ND	ND
1016	M	46	6.09	ND	ND
1017	M	58	6.17	*Naïve*	NA
1018	M	35	6.31	*Naïve*	NA
1019	M	46	6.39	*Naïve*	NA
1020	M	40	6.08	*Naïve*	NA
1021	M	67	5.79	ND	ND
001	M	51	6.37	PR-PRT	NR-NR
002	F	53	3.75	*Naïve*	NA
003	F	45	4.30	*Naïve*	NA
004	F	71	ND	*Naïve*	NA
005	F	52	8.09	*Naïve*	NA
006	M	45	7.00	PR	NR
007	F	35	8.00	*Naïve*	NA
008	F	51	ND	*Naïve*	NA
010	F	34	4.85	PR	NR
012	M	45	6.65	*Naïve*	NA
013	M	64	4.70	PR	NR
014	M	51	5.39	PR-PRT	NR-NR
015	M	30	ND	*Naïve*	NA
016	F	63	6.29	*Naïve*	NA
018	M	47	ND, 6.20	PR, SOF/LDV	NR, Relapse [e]
020	M	40	6.00	PR	NR
021	M	32	6.01	*Naïve*	NA
022	M	49	6.24	PR	NR
024	M	36	ND	*Naïve*	NA
025	M	48	6.90	PR	NR
026	M	59	ND	IR	NR
027	M	54	6.30, 7.10	PR, SOF/LDV	NR, Relapse [e]
028	M	42	4.36	PR	NR
030	M	37	5.30	*Naïve*	NA
031	M	45	ND	PR	NR
032	M	ND	5.10	*Naïve*	NA
034	F	58	5.60	PR	NR
036	F	54	5.30	*Naïve*	NA
039	M	43	6.49	PR	NR

a Viral load at time of sample collection. Log_{10} of viral load determined in international units per milliliter (IU/mL) with commercial kits COBAS®TaqMan HCV Test or Real Time HCV (Abbot), v2.0. <1.40 indicates detectable values, yet not quantifiable.

b **PR**: peg-IFN-α/ribavirin; ***Naïve*:** no treatment received; **PRT:** PR+telaprevir; **SOF/LDV:** sofosbuvir/ledipasvir; **IR:** IFN- α/ribavirin

c **NR:** Non-responder; **SVR:** Sustained virological response

d Treatment suspended due to intolerance to interferon

e 2 samples from the same patient were collected, after non-response to PR and after relapse to SOF/LDV

ND: Not determined or unknown
NA: Not applicable

Supplementary Table S2. GenBank accession numbers of HCV full-genome sequences corresponding to the 8 genotypes described so far that were used for the NS5B phylogenetic tree.

Genotype	Subtype	Country of isolation	GenBank Accession Nr	Observations
1	1a	China	KC844049	
		United States	AF009606	H77 Strain, subtype 1a reference sequence
		United States	EF621489	
		United Kingdom	AJ278830	
	1b	Germany	AJ238799	Con1 strain, subtype 1b reference sequence
		Brazil	EF032892	
		Japan	D90208	
		Russia	AF176573	
	1c	China	KC844047	
		India	AY051292	
		Indonesia	D14853	
2	2a	Japan	AB047639	
	2b	Denmark	JQ745651	
		Japan	AB559564	
	2c	France	KC197227	
	2i	Viet Nam	DQ155561	
	2k	Rep. Of Moldova	AB031663	
3	3a	China	KC844041	
		India	GQ275355	
		New Zealand	D17763	NZL1 strain, subtype 3a reference sequence
	3b	China	JQ065709	
		China	KC844044	
	3k	Canada	JF735122	
		India	D63821	
4	4a	Egypt	GU814265	
		Japan	AB795432	
	4v	Uganda	MH742365	
5	5a	China	KC844046	
		United Kingdom	Y13184	incomplete 5'NCR
6	6a	Viet Nam	EU246930	
	6b	Thailand	D84262	
	6d	Viet Nam	D84263	
	6f	Thailand	DQ835760	
	6h	Viet Nam	D84265	
	6k	China	DQ278891	
	6v	China	EU798760	
7	7a	Canada	EF108306	
	ND	Democratic Republic of Congo	MH940291	
8	ND	Canada	MH590701	
		Canada	MH590700	

ND: Not determined or Not assigned

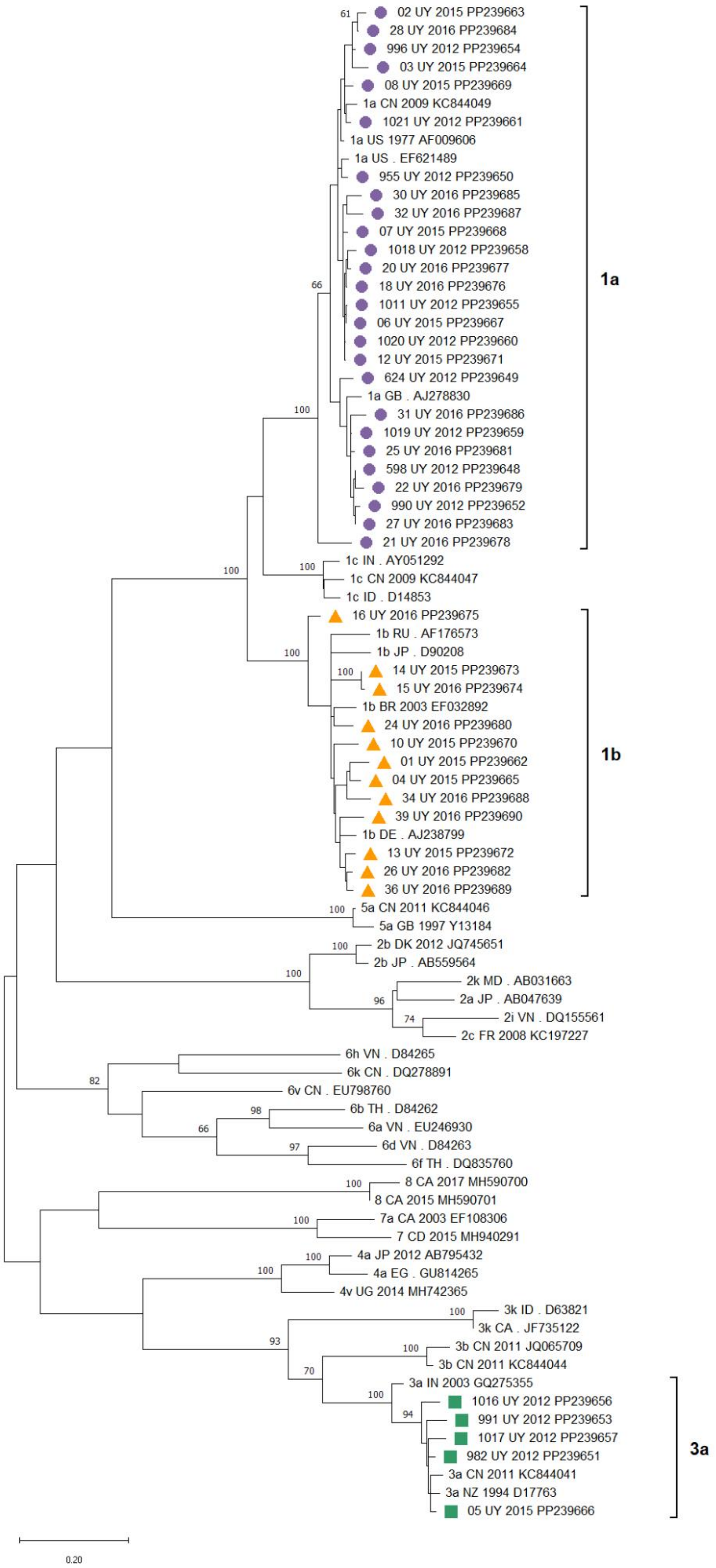
02 UY 2015 PP239663
28 UY 2016 PP239684
996 UY 2012 PP239654
03 UY 2015 PP239664
08 UY 2015 PP239669
1a CN 2009 KC844049
1021 UY 2012 PP239661
1a US 1977 AF009606
1a US . EF621489
955 UY 2012 PP239650
30 UY 2016 PP239685
32 UY 2016 PP239687
07 UY 2015 PP239668
1018 UY 2012 PP239658
20 UY 2016 PP239677
18 UY 2016 PP239676
1011 UY 2012 PP239655
06 UY 2015 PP239667
1020 UY 2012 PP239660
12 UY 2015 PP239671
624 UY 2012 PP239649
1a GB . AJ278830
31 UY 2016 PP239686
1019 UY 2012 PP239659
25 UY 2016 PP239681
598 UY 2012 PP239648
22 UY 2016 PP239679
990 UY 2012 PP239652
27 UY 2016 PP239683
21 UY 2016 PP239678
1a
1c IN . AY051292
1c CN 2009 KC844047
1c ID . D14853
16 UY 2016 PP239675
1b RU . AF176573
1b JP . D90208
14 UY 2015 PP239673
15 UY 2016 PP239674
1b BR 2003 EF032892
24 UY 2016 PP239680
10 UY 2015 PP239670
01 UY 2015 PP239662
04 UY 2015 PP239665
34 UY 2016 PP239688
39 UY 2016 PP239690
1b DE . AJ238799
13 UY 2015 PP239672
26 UY 2016 PP239682
36 UY 2016 PP239689
1b
5a CN 2011 KC844046
5a GB 1997 Y13184
2b DK 2012 JQ745651
2b JP . AB559564
2k MD . AB031663
2a JP . AB047639
2i VN . DQ155561
2c FR 2008 KC197227
6h VN . D84265
6k CN . DQ278891
6v CN . EU798760
6b TH . D84262
6a VN . EU246930
6d VN . D84263
6f TH . DQ835760
8 CA 2017 MH590700
8 CA 2015 MH590701
7a CA 2003 EF108306
7 CD 2015 MH940291
4a JP 2012 AB795432
4a EG . GU814265
4v UG 2014 MH742365
3k ID . D63821
3k CA . JF735122
3b CN 2011 JQ065709
3b CN 2011 KC844044
3a IN 2003 GQ275355
1016 UY 2012 PP239656
991 UY 2012 PP239653
1017 UY 2012 PP239657
982 UY 2012 PP239651
3a CN 2011 KC844041
3a NZ 1994 D17763
05 UY 2015 PP239666
3a
0.20

Supplementary Figure S1. Maximum likelihood phylogenetic tree of HCV partial NS5B sequences. The evolutionary history was inferred by using the Maximum Likelihood method and General Time Reversible model + Gamma distribution (5 categories (+*G*, parameter = 0.9431)) + invariable sites ([+*I*], 15.35% sites). Numbers at each branch of the tree show bootstrap support values ＞60%. The tree is drawn to scale, with branch lengths measured in the number of substitutions per site. This analysis involved 83 nucleotide sequences: 40 reference sequences for each HCV genotype and main subtypes (labeled with genotype, 2-letter country-code, year of isolation and GenBank Accession No) and 43 Uruguayan samples (labeled with strain nr, UY, year of isolation and GenBank Accession No). Uruguayan samples are indicated with purple dots (subtype 1a), orange triangles (subtype 1b) and green squares (subtype 3a). There were a total of 316 positions in the final dataset. Evolutionary analyses were conducted in MEGA11.0.13[45].

(A) Sample 1021 - major genotype 1a

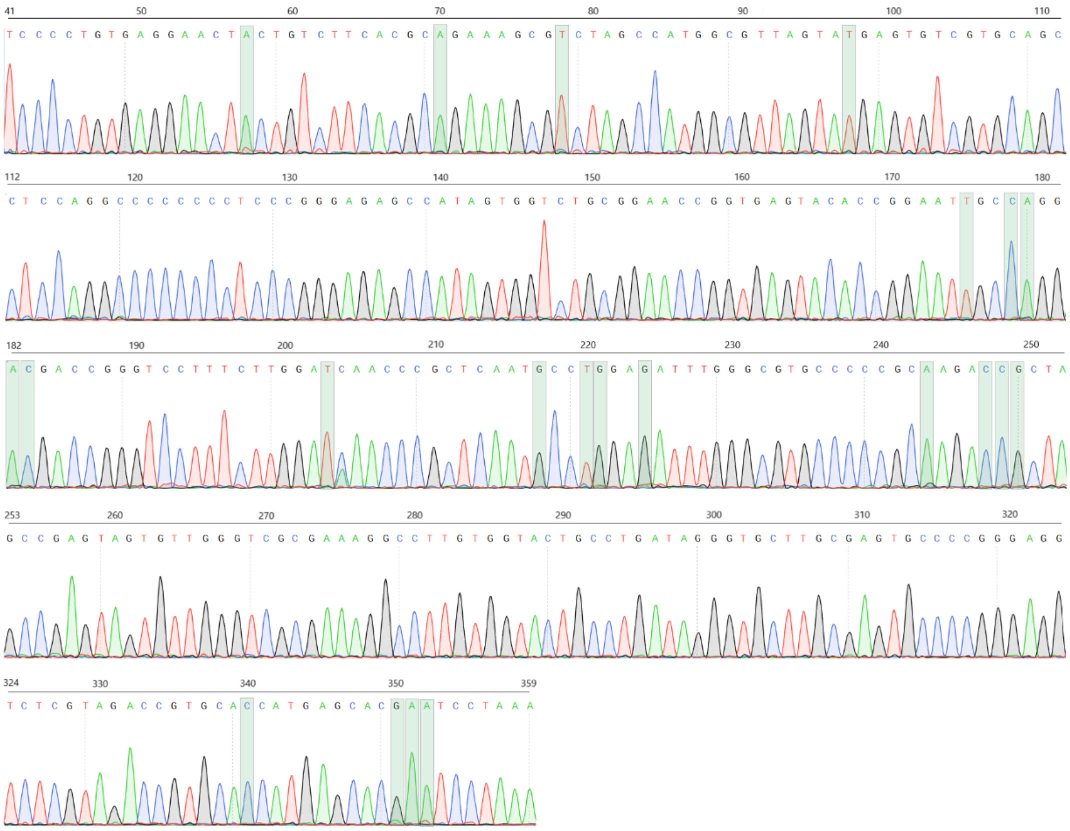

(B) Sample 05 - major genotype 3a

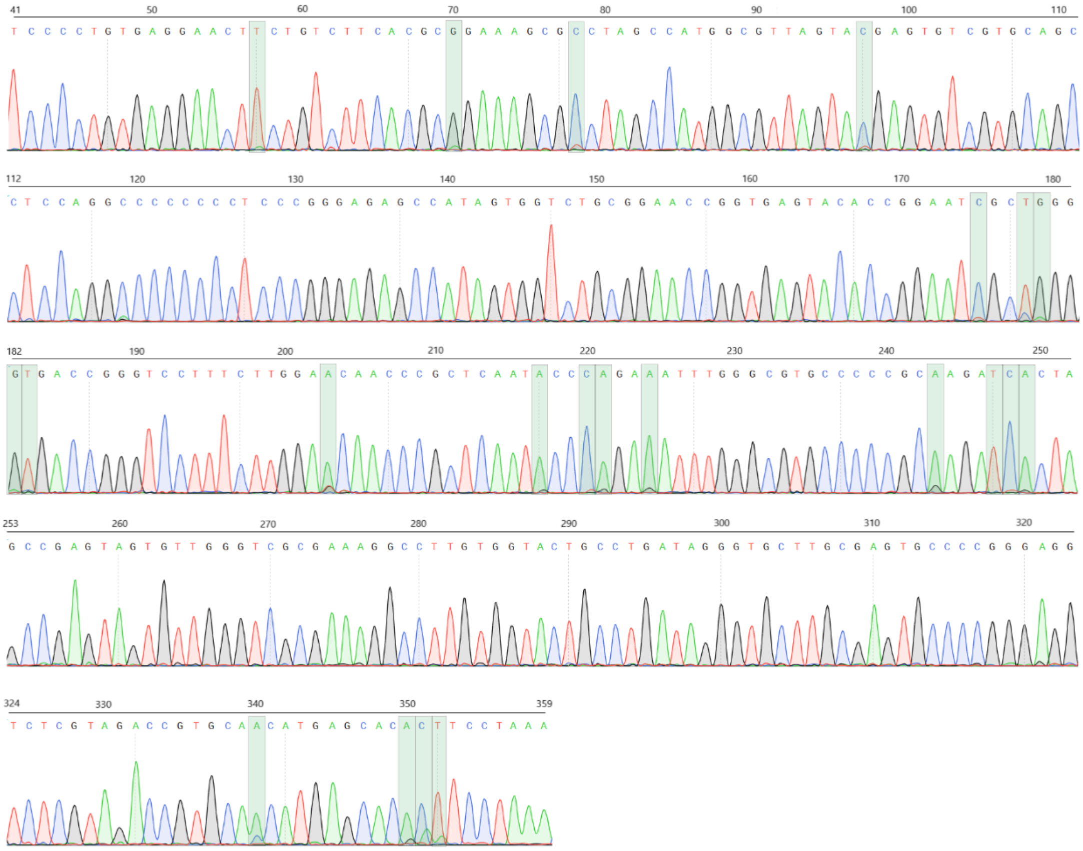

Supplementary Figure S2. Sanger sequencing electropherograms of IRES from samples with suspected mixed infections. A) Sample 1021 - major genotype 1a. B) Sample 05 - major genotype 3a. Full IRES region is shown, including 5'NCR from position 41 onwards and core region up to position 18 (nucleotide 359 of the genome). Numbering is according to strain H77 (reference sequence for genotype 1a). All positions highlighted in green indicate polymorphic sites between genotype 1a and 3a within the 5'NCR or the core regions. Double peaks suggesting possible mixed infections are more pronounced in sample 05, in agreement with NGS frequencies being higher for the minor genotype.

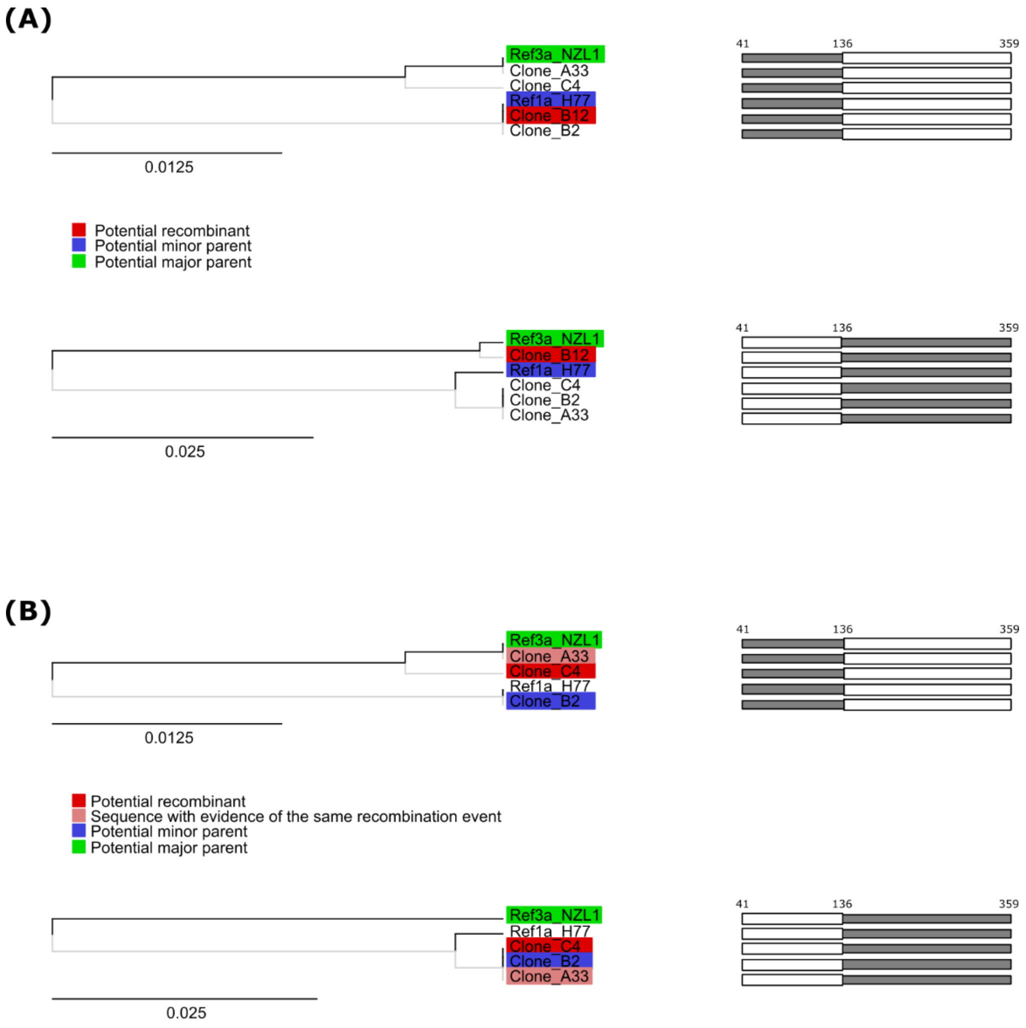

Supplementary Figure S3. UPGMA phylogenetic tree of HCV putative recombinant clones derived from RDP4.101 software analyses. Putative recombinant clone B12 **(A)** and C4/A33 **(B)** phylogenetic trees generated with the UPGMA agglomerative (bottom-up) hierarchical clustering method with default settings using RDP4.101 [55]. Top trees correspond to 5' portions of the IRES (nucleotides 41 to 135) and are indicated by grey bars on the right of each top tree. Bottom trees correspond to 3' portions of the IRES (nucleotides 136 to 359) and are indicated by grey bars on the right of each bottom tree. Potential major parents are highlighted in green, potential minor parents are highlighted in blue and putative recombinants in red and orange.
